# The caCORE Software Development Kit: Streamlining construction of interoperable biomedical information services

**DOI:** 10.1186/1472-6947-6-2

**Published:** 2006-01-06

**Authors:** Joshua Phillips, Ram Chilukuri, Gilberto Fragoso, Denise Warzel, Peter A Covitz

**Affiliations:** 1 Science Applications International Corporation, Annapolis, MD, USA; 2 Oracle Corporation, Reston, VA, USA; 3 National Cancer Institute Center for Bioinformatics, 6116 Executive Blvd., Rockville, MD 20852 USA; 4 SemanticBits LLC, Baltimore, MD, USA

## Abstract

**Background:**

Robust, programmatically accessible biomedical information services that syntactically and semantically interoperate with other resources are challenging to construct. Such systems require the adoption of common information models, data representations and terminology standards as well as documented application programming interfaces (APIs). The National Cancer Institute (NCI) developed the cancer common ontologic representation environment (caCORE) to provide the infrastructure necessary to achieve interoperability across the systems it develops or sponsors. The caCORE Software Development Kit (SDK) was designed to provide developers both within and outside the NCI with the tools needed to construct such interoperable software systems.

**Results:**

The caCORE SDK requires a Unified Modeling Language (UML) tool to begin the development workflow with the construction of a domain information model in the form of a UML Class Diagram. Models are annotated with concepts and definitions from a description logic terminology source using the Semantic Connector component. The annotated model is registered in the Cancer Data Standards Repository (caDSR) using the UML Loader component. System software is automatically generated using the Codegen component, which produces middleware that runs on an application server. The caCORE SDK was initially tested and validated using a seven-class UML model, and has been used to generate the caCORE production system, which includes models with dozens of classes. The deployed system supports access through object-oriented APIs with consistent syntax for retrieval of any type of data object across all classes in the original UML model. The caCORE SDK is currently being used by several development teams, including by participants in the cancer biomedical informatics grid (caBIG) program, to create compatible data services. caBIG compatibility standards are based upon caCORE resources, and thus the caCORE SDK has emerged as a key enabling technology for caBIG.

**Conclusion:**

The caCORE SDK substantially lowers the barrier to implementing systems that are syntactically and semantically interoperable by providing workflow and automation tools that standardize and expedite modeling, development, and deployment. It has gained acceptance among developers in the caBIG program, and is expected to provide a common mechanism for creating data service nodes on the data grid that is under development.

## Background

caCORE is a framework for creating syntactically and semantically interoperable biomedical information services [1]. Systems developed using the caCORE methodology use the same approach to defining, registering, and adopting data and representation standards. Clients of those systems can therefore draw upon multiple sources using similar API calls, and can rely on semantic equivalence of the data retrieved. caCORE consists of several components. Enterprise Vocabulary Services (EVS), a description-logics based thesaurus and ontology management system, provides terminology development and hosting services. The cancer Data Standards Repository (caDSR), an ISO/IEC 11179 metadata registry [2], provides for reference metadata management and distribution. The Cancer Bioinformatics Infrastructure Objects (caBIO) module provided the model-driven architecture for data interfaces that has since been adopted by all caCORE components. The Common Security Module (CSM) provides for highly granular access control and authorization schemes. Complete documentation and updated information on caCORE can be found on the NCICB web site[3]. A number of biomedical informatics groups use caCORE for the management of data standards and to create interoperable genomic, translational, and clinical research applications. Examples of such applications include the Cancer Models Database[4], caWorkbench[5], Cancer Molecular Analysis Project[6], and Cancer Centralized Clinical Database[7].

One of the primary incentives for making the caCORE development tools and methodology available to the broader community was the advent of caBIG[8]. The caBIG program was launched in 2003 as a federated program of biomedical information system and tool development. caBIG spans multiple research domains, including basic biology, genomics, proteomics, clinical trials, tissue banking and pathology, and imaging. caBIG presently includes participants from 50 cancer centers as well as other government, commercial, and non-profit institutions enaged in cancer research and patient advocacy.

In order for the tools developed by different development groups in caBIG to become interoperable, members of the caBIG program defined several different levels of system compatibility and interoperability that can be achieved by meeting certain sets of requirements[9]. The requirements are grouped into four major categories of standards: Information Models; Common Data Elements; Terminology; and Programming Interfaces. By meeting the requirements in all four categories, a system is said to have achieved a particular level of compatibility. The levels have been defined and labeled, in ascending order of stringency, as "Bronze", "Silver", and "Gold". As of the writing of this article caBIG developers are aiming for Silver-level compatibility in their projects, as the Gold level is still being defined by the program participants. Therefore, a toolkit that simplifies construction of a Silver-compatible resource was needed.

caBIG Silver compatibility calls for data systems to provide a documented API that serves up data objects derived from a domain object model that has been expressed as a UML class diagram. All data elements described by the model must be registered in the NCI caDSR. Terminology used for the model and data elements must originate from a caBIG-approved vocabulary source.

We assessed the functionality provided by the caCORE system with respect to caBIG compatibility and determined that it meets Silver level compatibility itself. We further determined that caCORE resources combined with other open-source components could be leveraged to assist other caBIG developers in with constructing their own Silver-compatible information systems. We therefore set about to upgrade and package the tools we had used to create caCORE itself. The product of this effort, the caCORE SDK, now enables any group to reproduce our methodology for implementing a caBIG Silver-compliant resource.

## Implementation

We analyzed and compared a number of open-source and low-cost tools and utilities that would support the construction of a model-driven system that was grounded in a solidly defined semantic foundation. The caCORE 3.0 information architecture formed the basis for our approach, but we selected components from external sources in addition to those developed at the NCI. The components were assembled and documented to support a streamlined workflow that accepts an information model expressed in the Unified Modeling Language (UML) as a starting point[10]. The model is semantically annotated using concepts from EVS terminology services and registered in the caDSR metadata registry for retrieval as needed by applications. The model is then fed into a Java code and database generator that creates fully functioning middleware connected to back-end data storage services. The resulting APIs are uniform across all data classes in the model, and provide for runtime access to structured research data as well as the associated metadata describing the semantics and information model.

### UML modeling tool

**Figure 1:**
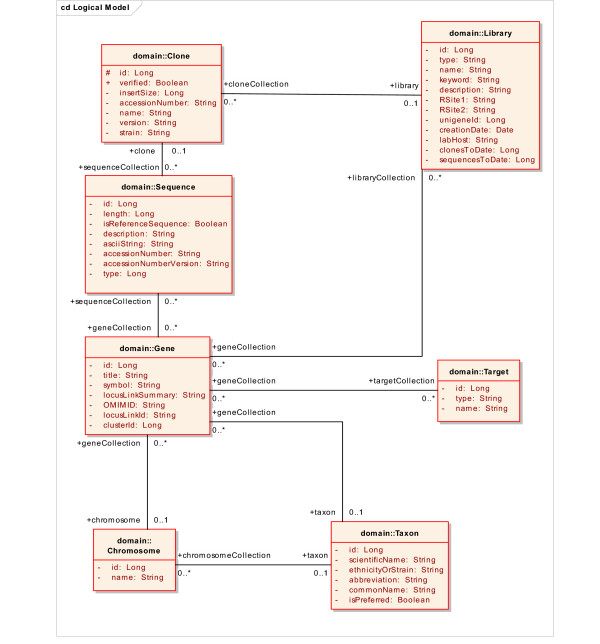
Example UML domain model, shown as a class diagram. The model shown here, a subset of the larger caBIO model used in the production caCORE system, includes several classes related to the class Gene. This model was used for initial testing and validation of kit components. After the kit was validated, the caCORE SDK was used with all the full-sized caCORE domain models (not shown).

UML can be used to model software, business processes and information models, including complex domain object models that represent data classes, attributes of each class, and class-class relationships. These class diagrams are electronically captured using a UML modeling tool. Several UML modeling tools – both commercial and open-source – are available in the market today. To select a UML modeling tool we conducted an extensive evaluation process based on a wide variety of selection criteria. The criteria included: ability to import/export UML models to and from XML Metadata Interchange (XMI) specification; support for all types of UML diagrams, for tagged values, and for stereotypes; performance; and cost. UML models must be exported to XMI format in order to be used in the subsequent steps in the caCORE SDK workflow[11]. Any UML 1.3 modeling tool that produces XMI 1.1 output should in principle be serviceable, though slight variations in XMI implementation may require some processing of the XMI files prior to usage with the caCORE SDK. Figure 1 shows an example class diagram from Enterprise Architect (EA), our first choice due to its relatively low cost and high performance[12].

### Semantic connector

Proper semantic integration requires that each class and class attribute from the UML model gets mapped to appropriate concepts in a controlled terminology. The caCORE SDK utilizes the NCI Thesaurus (NCIT) as its primary terminology source. The NCIT contains terms in use by the NCI and the cancer community, and is intended to facilitate interoperability, data sharing, and semantic integration between systems by mapping terms to unique concepts, providing codes, and providing base semantics and relations [13,14]. The NCIT is built in a description logics (DL) environment [13]. In the DL, concepts are compositionally defined by their placement in a subsumption hierarchy based on an agreed classification principle, and the role expressions asserted on the concept. Classification of the DL terminology by a reasoner can be considered a debugging step, and is utilized to validate defined concepts as well as identifying conflicting and inconsistent concepts. Although other terminology authoring systems can prove equally useful, we consider DL an integral part of terminology development because it helps to enforce the base semantics of the terminology. The rigour and consistency of the resulting product is deemed necessary to support the creation of metadata describing data that must be shared across disparate systems. In the future, other DL terminology sources in addition to the NCIT may be integrated with the caCORE SDK.

The concept selection process can be entirely manual, or it can be partially automated using the Semantic Connector, a tool supplied by the caCORE SDK. The Semantic Connector uses the UML Model expressed in XMI as input and uses the caCORE EVS APIs hosted at the NCI to search the NCI Thesaurus for appropriate concepts. The search algorithm specifically takes class and attribute names as input. String matching is conducted against the Preferred_Name and Synonym properties of the NCI Thesaurus. If an initial query with the whole UML class or attribute name fails to return results, the names are processed into component tokens based on underscores or case changes. For example, first_name or firstName would be split into the terms 'first' and 'name', and the semantic connector uses these component tokens to query the NCI Thesaurus.

The Semantic Connector returns a report as a comma-separated value file, listing possible matching concepts where found. This report is reviewed by developers and subject matter experts to ensure that the correct mapping of UML classes and attributes to semantic concepts. The report is edited manually to correct any errors in the automated output. In the cases where the automated matching fails to return a match, or returns more than one match to an existent concept, the assignment is made manually. Further, if the search and subsequent manual review reveals that no NCI Thesaurus concept (or combination of concepts) adequately represents the UML entity, suitable concepts are created. If a combination of concepts is required to adequately represent the class or attribute, they are explicitly ordered to preserve the intended semantics. The validated report containing mapped entities is then used to automatically insert tagged values for the semantic concepts back into the XMI representation of the UML model. The final product is thus an annotated UML model that includes all the semantic concept codes for each class and attribute. The model and the additional tagged values representing the semantic annotations can be viewed in the UML modeling tool.

### UML loader

The UML model, annotated with semantic concept codes, contains a considerable amount of metadata about the ultimate system that will be deployed. However, it is not in a form that is amenable to query and retrieval in a runtime environment nor easily queried by humans to make use of this information for other purposes such as creating forms to collect data. To address these limitations, we developed a strategy for transforming and loading the models into the caDSR, which provides APIs that support runtime access to metadata.

The caDSR is an implementation of the ISO/IEC 11179 standard for metadata registries, so we created a mapping between UML and ISO/IEC11179 at the metamodel level. To create the mapping, we experimented with various approaches and concluded that a UML *Class *is equivalent to an ISO/IEC 11179 *Object Class*, a thing in the real world; and an attribute of a UML class is equivalent to an ISO/IEC 11179 *Property*, a characteristic of a thing the real world. From there, other ISO/IEC 11179 metadata components, derived from Object Class and Property, can be formed. The joining of an Object Class and Property together forms an ISO/IEC 11179 *Data Element Concept*. A Data Element Concept, when joined to a *Value Domain*, forms an ISO/IEC 11179 *Data Element*.

Recognizing that UML modeling tools can export a model as an XMI file, we created the UML Loader, a utility that takes an XMI file as input. UML Loader uses mapping rules to convert UML Classes, Attributes, associations, and cardinality relationships into the corresponding ISO/IEC 11179 constructs: Object Class, Property, Data Element Concept, and Data Element. UML Loader then inserts these constructs into the caDSR. The insertion rules include a harmonization step in which the model components are compared to existing metadata components in the caDSR. Wherever an exact match to the same concept codes is found, the model component is not duplicated in the caDSR, and the existing component is re-used. In this way components of new models are automatically harmonized with those of existing models. We refer to the re-usable data element components as Common Data Elements (CDEs).

### Curation and specification of data standard constraints

The binding of specific thesaurus concepts to the UML model components and subsequent loading into the caDSR is necessary, but not entirely sufficient, to specify an interoperable system. The UML Loader automatically creates a default non-enumerated Value Domain for each Data Element. This default is sufficient for those Data Elements that do not need further specification beyond one of a general or primitive data type. However, for many fields, a crucial additional requirement is the specification of data standards that are to be used to constrain the allowable data entries in the final data management system. A list of enumerated permissible values or specific rules for values for a given Data Element needs to be explicitly defined such that values or rules can be retrieved and checked as needed during system instantiation or data validation.

UML provides the Object Constraint Language extension that could, in principle, be used this purpose. However, due to the level of interactive integration with EVS and existing caDSR content that is required to support construction of Value Domains, we have found that, in practice, it is more efficient to edit the Value Domains directly in the caDSR after UML model loading. For those Data Elements that require constraint by an enumerated list of values, data standards, or specific rules, further refinement of the model's metadata must be performed. The caDSR's CDE Curation Tool is a web application that enables a metadata curator with appropriate access permission to create or reuse existing Value Domains and insert or reference a list of permissible values. When using the CDE Curation Tool, the values may be drawn from EVS terminologies or other externally defined value sets. The CDE Curation Tool is not formally part of the caCORE SDK itself, but it is presently an essential auxiliary utility for this particular step in the workflow.

Final Curation of the model in caDSR includes a review using the caDSR tools and/or the APIs to ensure that all UML classes, attributes and associations have been properly transformed. A caDSR classification scheme equating to the UML Model is used to facilitate retrieval of all the associated caDSR metadata. After the model metadata has been reviewed for completeness, ensuring that all classes and attributes and appropriate associations have been created in caDSR as expected, additional information can be entered directly into caDSR using the caDSR tools, such as attaching a copy of the UML diagram for reference. In the final step of model curation, the model owner or metadata curator creates any necessary value enumerations or rules for Value Domains using the Curation Tool. The CDE Curation Tool is used to search for and incorporate EVS terminologies to build value lists based on existing data standards, thus allowing application owners to further disambiguate each Data Element.

### Code generator

Code generation reduces the cost of producing and maintaining large, consistent, and intuitive data-access APIs. The caCORE SDK includes a code generation framework called Codegen. Codegen is a complete code generation framework that is independent of any modeling tool and it can be used independently of the rest of the caCORE SDK.

Several existing, open-source, code generation tools were considered before it was deemed necessary to build Codegen. Among the tools considered were AndroMDA, AXgen, Eclipse Modeling Framework (EMF), and XDoclet [15-18]. The criteria used to evaluate these tools included the following: UML support; model API standardization; extent of model access; ease of extensibility; skill-set match; and extent of modeling constraints. While none of these tools, on their own, satisfied all the criteria, the components that some of these tools use can be combined in a way that does.

While Codegen is a complete framework, it is also rather lean in that it combines existing components in a useful way without introducing elaborate abstractions. Codegen uses the NetBeans Metadata Repository (MDR) to provide UML support, model API standardization, and full model access[19]. Maximal extensibility and minimal modeling constraints are achieved through Codegen's concepts of filter and transformation chains. Codegen utilizes Java-related skill-sets by using Java Emitter Templates (JET) as its primary template language[17]. The FreeMarker template language is also supported[20].

## Results

### Overview of the caCORE SDK

caCORE systems are built using the model-driven architecture paradigm, with substantial extensions added to support the incorporation of a much richer semantic framework. The development methodology is a modified version of the Rational Unified Process embedded within a lightweight version of the traditional waterfall approach[21]. Therefore, usage of the SDK follows a particular workflow pattern that mirrors our approach to constructing caCORE-type resources. The steps in the workflow include: Use case development; information modeling; semantic annotation; metadata registration; code generation; system deployment.

The result is a fully functioning multi-tiered system with a middleware layer that provides well defined APIs. The data classes, their attributes, and the data itself are tagged with appropriate concepts from a description-logics ontology. The ontology tags, associated definitions, and semantic relationships are all available in the runtime programming environment from the caCORE metadata registry, the caDSR.

The caCORE SDK includes numerous components (Table 1). Most are included with the kit download, including those open-source components that are from other projects. The kit also includes a comprehensive Programmer's Guide. Users must obtain their own UML 1.3-compliant UML modeling tool that can export XMI 1.1 or 1.2. And at present, the UML Loader utility that registers the model in the caDSR metadata registry can only be run in consultation with the caDSR support team. Future versions of the kit are expected to provide direct access to UML Loader.

**Table 1:** Components of the caCORE SDK.

**Component Name**	**Version**	**Description**	**URL**	**Incl.**
Java 2 Standard Edition (J2SE)	j2sdk1.4.2_06 or higher	The J2SE Software Development Kit (SDK) supports creating J2SE applications		No
UML 1.3 Modeling Tool that produces XMI 1.1 output	EA 4.50.744	We recommend using Enterprise Architect (EA)		No
Ant.jar	1.6.2	Apache Ant is a Java-based build tool		Yes
activation.jar		The classes that make up the JavaBeans Activation Framework (JAF) standard extension are contained in the included Java Archive (JAR) file, "activation.jar"		Yes
alteredHibernate2.jar		Modified Hibernate code decoupling SearchCriteria object from the Session Object packaged within Hibernate 2.1 classes.		Yes
aspectjrt.jar		Aspectj is a seamless aspect-oriented extension to the Java™ programming language		Yes
aspectjtools.jar		Aspectj contains a compiler, ajc, that can be run from Ant. Included in the aspectjtools.jar are Ant binaries to support three ways of running the compiler		Yes
axis-ant.jar		Ant tasks for building axis.		Yes
axis.jar		Apache Axis is an implementation of the Simple Object Access Protocol (SOAP)		Yes
cglib-full-2.0.1.jar	2.0.1	Dynamic JAVA byte code generator		Yes
codegen.jar		Classes required for JET template compilation.		Yes
commons-collections-2.1.jar	2.1	Apache Jakarta Commons utilities		Yes
commons-dbcp-1.1.jar	1.1	The Jakarta Commons DBCP Component provides database connection pooling.		Yes
commons-discovery.jar		Apache Jakarta Commons discovery utilities		Yes
commons-lang-1.0.1.jar		Provides a helper utilities for the java.lang API.		Yes
commons-logging-1.0.3.jar		Provides a helper utilities logging.		Yes
commons-logging.jar		Apache Jakarta Commons logging utilities		Yes
commons-pool-1.1.jar	1.1	The Jakarta Commons Pool Component provides a generic object pooling API.		Yes
datafile.jar		Java data file read/write utility that provides a convenient set of interfaces for reading and writing data to and from files in widely accepted format such as comma separated values (CSV), fixed width, tab separated, as well as others		Yes
db2java.jar		Contains classes to support connections to DB2 databases.		Yes
dom4j-1.4.jar	1.4	Contains classes that allow you to read, write, navigate, create and modify XML documents.		Yes
ehcache-0.7.jar	0.7	EHCache is a pure Java, in-process cache.		Yes
freemarker.jar		FreeMarker is a "template engine"; a generic tool to generate text output (anything from HTML or RTF to auto generated source code) based on templates.		Yes
hibernate3.jar	3.0	Hibernate 3.0 is used for the server-side ORM		Yes
Jakarta-oro-2.0.8.jar	2.0.8	The Jakarta-ORO Java classes are a set of text-processing Java classes that provide Perl5 compatible regular expressions, AWK-like regular expressions, glob expressions, and utility classes for performing substitutions, splits, filtering filenames, etc.		Yes
jalopy-1.0b11.jar	1.0b11	Source code formatter.		Yes
jalopy-ant-0.6.2.jar	0.6.2	Ant task for building jalopy.		Yes
jaxen-core.jar		The jaxen project is a Java XPath Engine. jaxen is a universal object model walker, capable of evaluating XPath expressions across multiple models.		Yes
jaxen-jdom.jar		The jaxen project is a Java XPath Engine. jaxen is a universal object model walker, capable of evaluating XPath expressions across multiple models.		Yes
jaxrpc.jar		Java API for XML-based RPC		Yes
jdom.jar	1.0	Java-based solution for accessing, manipulating, and outputting XML data from Java code.		Yes
Jdtcore.jar		Eclipse Tomcat Plugin		Yes
jetc-task.jar		An ANT task for translating JET templates outside of Eclipse		Yes
jmi.jar		JMI is a standards-based, platform independent, vendor-neutral specification for modeling, creating, storing, accessing, querying, and interchanging metadata using UML, XML, and Java.		Yes
jmiutils.jar		Part of the NetBeans MDR codebase. It contains general utility classes for doing things such as generating class files and querying MOF models. Most importantly, it contains the implementations of XMI readers and writers.		Yes
jta.jar		JTA specifies standard Java interfaces between a transaction manager and the parties involved in a distributed transaction system		Yes
junit-3.8.1.jar	3.8.1	JUnit is a regression testing framework that is used by the developer who implements unit tests in Java		Yes
junit.jar		JUnit is a regression testing framework that is used by the developer who implements unit tests in Java		Yes
log4j-1.2.8.jar	1.2.8	Log4j is an open source tool developed for putting log statements into your application. With log4j you can enable logging at runtime without modifying the application binary.		Yes
log4j.properties		Configuration file used by Log4J		Yes
mail.jar		JavaMail API		Yes
Mdrant.jar		Ant tasks for building MDR.		Yes
Mdrapi.jar		MDR implements the OMG's MOF (Meta Object Facility) standard based metadata repository and integrates it into the NetBeans Tools Platform. It contains implementation of MOF repository including persistent storage mechanism for storing the metadata. The interface of the MOF repository is based on (and fully compliant with) JMI (Java Metadata Interface – JSR-40).		Yes
mof.jar		Archive containing the classes pertaining to Meta-Object Facility (MOF) specification from Object Management Group (OMG).		Yes
mysql-connector-30.jar		Archive containing MySQL 3.0 JDBC driver classes.		Yes
nbmdr.jar		Extended implementation of the Meta-Object Facility, XML Metadata Interchange, and Java Metadata Interface standards.		Yes
Openide-util.jar	4.0	Contains low level basic support classes that MDR depends on.		Yes
osgi.jar	3.0	The OSGi™ specifications define a standardized, component oriented, computing environment for networked services.		Yes
resources.jar		Contains code central to the Eclipse platform. The caCORE SDK uses an the "jetc" Ant task to translate JET templates to Java source code. That Ant task uses code from the Eclipse Modeling Framework (EMF) plug-in to do the translation. The EMF plug-in depends on code in resources.jar		Yes
Runtime.jar		Archive containing the classes necessary to run applications developed on Eclipse Rich Client Platform (RCP).		Yes
saaj.jar		Archive containing the classes pertaining to SOAP with Attachments API for Java (SAAJ) specification. SAAJ is included in Java Web Services Developer Pack (JWSDP).		Yes
Saxpath.jar	1.0-FCS	SAXPath is an event-based API for XPath parsers, that is, for parsers that parse XPath expressions.		Yes
servlet.jar	J2EE 1.3	Archive containing the classes pertaining to J2EE servlet API specification.		Yes
uml-1.3.jar	1.3	Object Management Group (OMG) UML metamodel specification.		Yes
wsdl4j.jar		Web Services Description Language support for Java		Yes
xerces.jar		XML Parser		Yes
xercesImpl.jar	2.4.0	Xerces Java Parser		Yes
xml-apis.jar	2.0.2	XSLT processor for transforming XML documents into HTML, text, or other XML document types.		Yes
xmlrpc.jar		Apache XML-RPC is a Java implementation of XML-RPC, a popular protocol that uses XML over HTTP to implement remote procedure calls.		Yes

### Use cases

A use case is a structured textual representation of how users interact with a computer application. Use cases typically define one or more actors, a workflow, pre-conditions, post-conditions, and alternate scenarios. The caCORE software development process begins with use case development, using whatever tool the team prefers. The level of formality, granularity, and depth of the use cases can be adjusted based upon the needs and constraints of the project team. We stress that use case development should not be skipped, as use cases provide tangible, stable indicators for project requirements and thus help avoid drift and scope creep. Example use cases for a number of projects can be found on the NCICB web site.

### Information modeling and semantic annotation

Once a sufficiently comprehensive set of use cases has been collected, the next step is to create a domain model in UML. This model will serve as the primary determinant of the information and programming interfaces that are produced, and is also the basis for generation of the actual software code. Classes in the model should represent discrete scientific entities, and attributes of the classes should represent specific characteristics of the entities. The attributes become Data Elements in a software system. For example, a class might be defined as 'Gene', and an attribute of that class defined as 'Symbol' (Figure 1). In addition to classes and attributes, the model also specifies class-class associations including cardinality and direction. It is imperative that the UML model be annotated with descriptions; this facilitates the subsequent semantic integration, where UML entities (classes and attributes) are matched to vocabulary concepts, and the matches require review by a subject matter expert.

After the classes and attributes in the UML model are defined, but before it is finalized, the modeler undertakes the process of semantic integration, which results in the annotation of the UML entities with concept codes from a controlled terminology system. In practice this involves using the Semantic Connector utility, included with the caCORE SDK, to tag data classes and attributes with concepts the NCI Thesaurus. This utility accepts an XMI representation of a UML class diagram as input, compares the class and attribute names to the NCI Thesaurus, and returns a report of matches between the class and attribute names and NCI Thesaurus concepts, along with concept names, codes, preferred terms and definitions.

**Figure 2:**
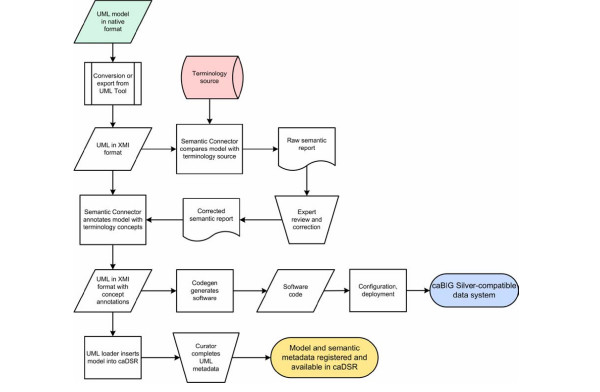
caCORE SDK Workflow. A UML object model and a description-logic terminology source (NCI Thesaurus in the present work) are the inputs into the workflow. The model is exported from the format native to the tool it was developed in to the standard XMI representation. The XMI file is then annotated with terminology concepts using Semantic Connector. The annotated model is used as input into Codegen, which generates the software for a caBIG-compatible data system with object-oriented APIs. The annotated model is loaded as metadata into the caDSR using UML Loader. Model metadata is reviewed and completed by a curator using caDSR utilities, and then becomes available from the caDSR APIs and web applications.

The report is reviewed by a subject matter expert who makes appropriate edits and selections for the final concept associations using a text editor or spreadsheet software. Table 2 shows a portion of the Semantic Connector report for the *Target *class and its attributes after manual review and annotation. In many cases the expert will assess whether a UML entity is best represented by a single concept or a combination of concepts. Consultation with an authorized editor of the terminology source may be warranted. Once the report has been appropriately edited to contain the correctly ordered concept mappings, including names and codes and definitions, it is fed back into the Semantic Connector utility, along with the original model, to produce a revised XMI file that now includes all of the relevant ontology concept information from the terminology source. This modified file can be re-loaded into the UML modeling tool as needed. Once the model has been finalized, it is fed into the next steps in the workflow (Figure 2).

**Table 2:** Semantic Connector report for the *Target *class and its attributes.

**UML Entity**^‡^	**Concept Name**	**Concept Preferred Name**	**Classification**	**Concept Code**	**Concept Definition**	**Concept Definition Source**	**Human Verified**
Target	Candidate_Disease_Gene	Candidate Disease Gene	ObjectClass	C19389	A gene proposed to have a primary role in a disease, based upon its known function in other organisms or model systems or based upon its physical proximity to markers linked to a genetic disease.	NCI	y
id	Identifier	Identifier	Property	C25364	One or more characters used to identify, name, or characterize the nature, properties, or contents of a thing.	NCI	y
type	Type	Type	Property	C25284	A subdivision of a particular kind of thing.	NCI	y
name	Name_Generic_Concept	Name	Property	C42614	The words or language units by which a thing is known.	NCI	y

^‡^A class or an attribute of a class

### UML model loading

The semantically annotated version of the model is exported in XMI and used as an input to UML Loader, a Java application that performs the transformation of metadata from the model into caDSR metadata (Figure 2). UML model metadata corresponding to various elements of a class diagram is transformed into caDSR metadata by creating, or finding and reusing, the different types of caDSR metadata components. A high level ISO/IEC 11179 Classification Scheme is created for each UML model and used to classify and group all the model metadata, simplifying identification and retrieval via caDSR tools and APIs.

We used the UML Loader to insert components from a semantically annotated version of the model shown in Figure 1 into the caDSR. In the case of the UML class "Chromosome", the following caDSR entities are created: an Object Class with a long name of "Chromosome"; Properties "Identifier" and "Name" corresponding to attributes "id" and "name"; Data Element Concepts "Chromosome Identifier" and "Chromosome Name" ; Data Elements "Chromosome Identifier java.lang.Long" and "Chromosome Name java.lang.String", which include the default Value Domains for the Long and String data types, respectively. In the case of this example, the default Value Domains were left unmodified.

Details of the class-class associations are recorded as relationships between caDSR Object Classes, including cardinality and direction. Inheritance associations are treated as an "IS_A" relationship and transformed in a manner such that the class attributes associated with the target class are inherited by the source class. The resulting loaded metadata can be retrieved from the caDSR APIs or viewed interactively with the CDE Browser web application. The UML model shown in Figure 1 was loaded in this manner and can be viewed in the CDE Browser web application in the caCORE Context.

### Code generation

After the UML model has been curated, Codegen is used to produce code and other artifacts from elements of the model. The code generation process is an automated workflow that is directed by an XML control file. The two steps in that process include first selecting elements of the model using a 'filter-chain' and then generating artifacts from those elements using a 'transformer-chain'. A filter-chain can select model elements that, for example, have names that will match a given regular expression or that are members of a class hierarchy. A transformer-chain consists of one or more 'transformers' that will generate some artifact from each model element that is selected by a filter-chain. The type of transformer used depends on the type of artifact desired. The caCORE SDK supplies JET templates as the transformers that produce Java code. The syntax of JET templates is a subset of Java Server Pages (JSP) syntax, which makes writing templates easy for those with Java skills.

When a template is executed, Codegen passes it a reference to the selected model element. The template code can then inspect the properties of that element to determine how to generate code from it. For example, the code can determine the element's name, attributes, or any ontological information added by the Semantic Connector utility. The references passed to templates are Java objects that implement standard interfaces, as specified by the Java Metadata Interface (JMI) standard. For example, if the model element is a UML 1.3 class, then the object would implement the 'org.omg.uml.foundation.core.UmlClass' interface. From any model element reference, the transformer code can navigate to all other information in the model by using the JMI interfaces alone, but Codegen provides utility classes for performing common operations, such as retrieving all classes associated with a particular class. Out of the box, the caCORE SDK provides transformers to generate Java Bean implementations of UML class elements; Hibernate Object-Relational mapping files; and other configuration files needed by the caCORE architecture.

### Object to relational mappings

The caCORE SDK generates the implementation of an object-oriented API to a relational data source. Object-oriented queries created by clients are sent to the persistence layer, where they are mapped into equivalent SQL statements. The resulting database records are used to populate the caCORE domain objects that are sent back to the client. The translation of domain objects to database records, and back, is handled by the open-source object-relational mapping tool Hibernate[22]. To perform this translation, Hibernate needs XML files containing the metadata that describes how classes map to tables, how attributes map to columns, and how associations between classes map to relations between tables. Codegen generates these XML files from the UML model.

According to the caCORE SDK's model-driven process, the modeler creates a UML data model within the same model that contains the domain object model. The data model describes the mappings between the object model and a physical relational schema. This approach works for both new and existing database schemata. There are various approaches to implementing object-relation mapping, each with its own trade-offs and implications for physical database structure. Hibernate can accommodate most approaches. However, the Codegen transformers that generate the Hibernate XML metadata files follow a single approach which places constraints on the database structure. For example, it is assumed that all tables will use a surrogate primary key. The details of these constraints are explained in the caCORE SDK's Programmers Guide.

### Database creation

After the data model containing the mapping between object model and physical relational schema is created, data definition language (DDL) scripts containing SQL commands for creating database schema could optionally be generated using the "Generate DDL" utility in EA. These SQL scripts have to be executed in the chosen RDBMS environment to actually create the necessary database schema objects such as: tables and views, and database constraints such as: primary keys, unique keys, and foreign keys. This exercise can totally be skipped when using an existing/legacy database. Essentially, caCORE SDK supports creation of object-oriented data access API for two types of systems: systems with existing databases and brand new systems.

During model transformation the UML Loader creates an alternate name for each Data Element in caDSR corresponding to the fully qualified attribute name. If the caCORE SDK naming convention is followed, these alternate names will match the mapping attribute names used to associate data model tables and fields with domain object model classes and attributes, forming the basis for easily identifying the caDSR Data Elements associated with the data model. For example, if the data model describes a table and field mapped to the attribute "name" of the "Chromosome" class of the domain object model, the alternate name "gov.nih.nci.cabio.domain.Chromosome.name" is created and can be used as a search term to find the Value Domain list or rules associated with this Data Element.

### System architecture and deployment

**Figure 3:**
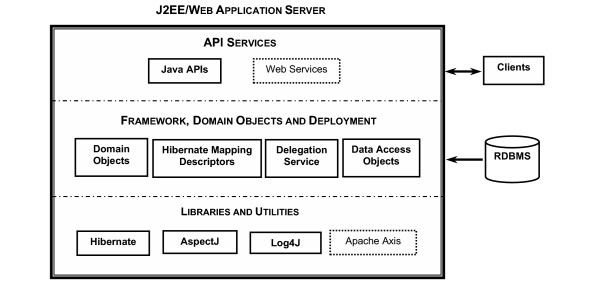
Architecture of caCORE SDK-generated system. Systems developed using caCORE SDK are deployed as a Web Application Archive (WAR) to a J2EE application server such as JBoss or a web application server such as Tomcat. Contents of the WAR file can be logically grouped into three categories. Libraries and Utilities are typically packaged as JAR files, and include AspectJ for auditing, Log4J for logging, and Hibernate for object-relational mapping. Framework, Domain Objects and Deployment Descriptors include caCORE SDK classes needed at runtime, domain objects with corresponding hibernate mapping files generated by caCORE SDK, and property files containing configuration parameters. API Services function as a façade or an entry point to the system. Client requests are processed by the interface proxy in the application server and mapped to the appropriate data source by the delegation service. In version 1.0.2 of caCORE SDK, described in this article, only Java APIs are generated; in subsequent versions Web Services APIs can also be generated, with Apache Axis providing SOAP message support.

The default output of the caCORE SDK is a fully functional, data-access system that is similar to the caBIO module of caCORE 3.0 (Figure 3). The system is composed of six components that function on three tiers. The components include domain objects, a query API, the interface proxy, a delegator/service locator, internal data source, and external data source.

The domain objects are Java classes representing biomedical concepts, like Gene or Protein. Instances of these classes are populated with data by components in the data source tier and then sent by components in the server tier to the client tier. These classes follow a Java Bean-like pattern, which means that the properties of each object are accessible through operations whose names begin with "get". For example, to retrieve the collection of Library objects associated with a Gene object, the client invokes the getLibraryCollection operation on the Gene object.

The query API provides both a simple, query-by-example metaphor and a more powerful search criteria model. In the query-by-example approach, the client constructs a domain object and populates it with property values that are similar to those of objects that should be retrieved. The search criteria model provides a 'detached' version of the popular Hibernate 2 Criteria API, which enables expression of complex query constraints. The implementation of the Hibernate 2 Criteria API has been modified (detached) to allow it to run on the client tier without making database connections to the data source tier. The query API includes a utility class, called ApplicationService, which sends queries to the caCORE server tier.

The interface proxy component provides a common entry-point into the caCORE server-tier for all types of caCORE clients. The implementation of the interface proxy that is provided with the caCORE SDK is a Java Servlet that services clients using HTTP tunneling. The client, for example, ApplicationService, packages a query within a caCORE Request object and writes it (using Java Object Serialization) over an HTTP connection to the interface proxy servlet, which deserializes the HTTP request payload back into a caCORE Request object. The results of a query are written back to the connection and deserialized by the client.

The delegator passes query requests from the interface proxy, to the logical data source that is responsible for the set of domain objects being queried. The service locator uses service metadata to map data sources to a particular implementation of a logical data source. The caCORE SDK comes with implementations for two data sources: ORM and ExternalSystem. The ORM implementation uses Hibernate to translate object criteria into SQL statements, execute those statements in a relational database, and populate domain objects from the returned records. The ExternalSystem implementation simply forwards the request to another interface proxy.

Client tier components run as (or within) standalone Java applications. The client installation involves only putting the client jar (Java Archive) file, and a few other jar files, on the Java runtime classpath. Server- and data source tier components run within an application server. Since the interface proxy component of the system is implemented as a Java Servlet, the server can be installed in any application server that supports the Java Servlet 2.3 specification. By default, the caCORE SDK will download and deploy the server to Tomcat, which is a free and open-source application server that is also the reference implementation of the Java Servlet specification.

### Systems built using caCORE SDK

A number of biomedical research information systems have been constructed or extended using caCORE SDK. The caCORE 3.0 suite of components was built using caCORE SDK to create the object-oriented middleware and API interfaces to the various underlying data resources [3]. caCORE 3.0 provides runtime data services for terminology, metadata, and biomedical data. The SNP500Cancer project is specifically designed to generate resources for the identification and characterization of genetic variation in genes important in cancer [23]. SNP500Cancer has used the caCORE SDK to add publicly accessible object-oriented APIs to their database resource. The Protein Information Resource (PIR), an integrated public resource of protein informatics that supports genomic and proteomic research and scientific discovery, created a new object model in UML to represent their information structures, and then used the caCORE SDK to provide interoperable APIs [24]. The in vivo Image Repository (I3) aims to provide access to imaging resources that will improve the use of imaging in today's cancer research and practice by: increasing the efficiency and reproducibility of imaging to support cancer detection and diagnosis, leveraging imaging to provide an objective assessment of therapeutic response, and ultimately enabling the development of imaging resources that will lead to improved clinical decision support [25]. I3 used the caCORE SDK to construct its entire back-end and middleware. Development using caCORE SDK is currently underway in several other projects affiliated with caBIG.

## Discussion

A number of software development tools are emerging that support specific needs of biomedical informatics. The majority, however, have been created to support data analysis and manipulation use cases, and not semantics-based data modeling and management. The caCORE SDK fills this gap by providing a well structured approach to linking information models that represent data services with DL semantic formalisms. The resulting n-tier system that is automatically instantiated provides a basis for federated interoperability across all sites that use the same semantic model and APIs.

The caCORE SDK relies on UML to represent its domain information models. UML is a maturing standard that has gained widespread acceptance in the software industry. Its provision for graphical representations of models makes it a fine choice for working with biomedical subject matter experts. Since every UML model conforms to the same underlying meta-model, these models are readily leveraged to generate consistent, well-structured software. Thus the caCORE SDK provides a full realization of the model-driven architecture paradigm. The model is the code, the code is the model.

For many years the only available UML modeling tool was a costly product that was not widely accessible to modestly funded developers in the biomedical informatics community. There are now several low cost choices and an open-source alternatives. We selected Enterprise Architect for use with the caCORE SDK due to its combination of low cost and high performance. Poseidon for UML and ArgoUML are notable alternatives[26,27]. Processing the XMI that is exported from a given tool is necessary, as each vendor implements the XMI standard slightly differently. The XMI processor for EA-exported models is included in the caCORE SDK.

Our requirements for semantic expressiveness and runtime services demanded that we extend the basic UML modeling process typically used in business software development. We were able to ground the models in a richer DL semantic structure without sacrificing the benefits of the model-driven architecture paradigm by mapping each UML data class and attribute to concepts in the NCI Thesaurus, a concept oriented thesaurus with ontology-like properties. The Semantic Connector provides a modest degree of automation to this added procedure, minimizing the burden on the software development team. The UML loader captures the resulting annotated model and registers it as metadata in the caDSR. The caDSR provides tools to specify permissible value constraints for the Data Elements in the model. The caDSR also provides runtime access to metadata that can be used to satisfy a variety of discovery, semantic comparison, and data validation use cases.

Automated code generation tools have been available for a variety of programming languages for a number of years. The quality and ease of use of such tools has varied widely. Until recently the best alternatives were often quite costly. Our approach couples the meta-model structures in the XMI representation of a UML model with a template that dictates what the final code will look like. Crucial to this our approach was the availability of a number of open-source utilities, in particular the NetBeans MDR, that made it straightforward to programmatically manipulate UML object models. We were able to efficiently couple and synchronize the model with the resulting software. The code generation itself takes only minutes, freeing the development team to focus on the scientific issues and associated semantics that impact the model structure.

The semantically connected UML models in their native and XMI formats are sufficient to support the software development team that is using the SDK for its own purposes. However, these file formats are not optimal to satisfy use cases that demand model query and retrieval services. Developers that wish to query and retrieve information about specific data classes, attributes, relationships and definitions within the models need a runtime model management environment that supports such functionality. We found the most straightforward way to satisfy such requirements was to map the UML meta-model components to ISO/IEC 11179 and create a specialized loading utility that implements these mappings and loads models as metadata components in the caDSR. Thus the caDSR supports granular query and retrieval functions that enable application developers to present the definitional metadata about the model in appropriate user interface components.

The caCORE SDK has been used successfully by a number of projects at the NCI and in the broader community affiliated with the caBIG program. We are presently tying these various caCORE SDK-generated systems together in a common data and analysis grid framework[28]. In this way we expect to provide a single point of entry for the advertising, discovery, and invocation of caBIG-compatible resources.

## Conclusion

The caCORE SDK was constructed to support both internal development at the NCICB as well as community developers in the caBIG program. The caBIG Vocabulary and Common Data Elements workspace has decided that UML modeling, caDSR metadata registration, and terminology standards must be implemented in caBIG 'Silver' compatible systems. Thus the caCORE SDK provides a relatively straightforward development path towards Silver-level compatibility.

The caBIG Architecture workspace is currently defining a data grid framework that will become the caBIG 'Gold' standard for interoperability. This grid architecture – named 'caGrid' – will include a service registry for advertising Gold-compatible data and analytical services that are on caGrid. The advertised metadata will originate from the output of the caCORE SDK. Thus we expect to create a clear mechanism for systems that have achieved Silver compliance to add the necessary adaptors and extensions to enable them to advertise their services and become part of the caGrid federation.

As this manuscript was being completed, the next version of the caCORE SDK was released, and included support for integrating the caCORE 3.0 Common Security Module into the target architecture. This feature enables secure access control to systems generated by the caCORE SDK, and adds a standardized write-API that permits applications using the system to insert and persist data objects in addition to reading them. The new version also supports the generation of a SOAP web services API in addition to the Java API.

While the motivation behind the caCORE SDK originated in the area of cancer informatics, the approach and tools can be applied to any domain of biomedical research. We expect that the caCORE SDK will lower the barrier to developing and deploying model-driven systems that are integrated with structured semantics in many different areas.

## Availability and requirements

• Project name: caCORE Software Development Kit

• Project home page: . This page has links to the latest version, which readers are encouraged to use. Version 1.0.2, described in this article, can be found at .

• Operating system: Platform independent

• Programming language: Java, JET

• Other requirements: Tomcat 4.1.31, mySQL 4.1.9, Ant 1.6.2, UML 1.3/XMI 1.1-compliant modeling tool. Other software components, most included with SDK, that are listed in Table 1.

• License: caCORE Software Development Kit License, Version 1.0, which applies equally to academic and non-academic users, and can be found at .

The following are the system requirements for deploying and running an application generated using caCORE SDK:

• Operating system: Platform independent

• JDK 1.4.2_06 tested.

• Web container such as Tomcat 4.1.31 or J2EE application server such as JBoss 4.0.

• caCORE SDK comes configured to use mySQL for automated database generation. Oracle, DB2 and other databases can also be used with several configuration adjustments.

## Competing interests

The author(s) declare that they have no competing interests.

## Authors' contributions

JP was responsible for the overall design and construction of Codegen, and wrote the Codegen sections of this article. DW and RC conceived of the design to transform UML metamodel structures expressed in XMI into ISO/IEC 11179 metadata, and contributed to the UML Loader section of this article. RC was the lead developer and architect for the UML Loader. DW is the technical manager and RC the lead developer for the caDSR system. GF lead the development of the format and content of semantic connector report for EVS review and helped work out the conventions for naming and ordering the mapped concepts. DW contributed to the design of the semantic connector report format so that the entities could be properly annotated and ordered for processing by the UML Loader. PC conceived of the high-level design of the caCORE SDK and wrote and edited most of this article. All authors read and approved the final manuscript.

## Pre-publication history

The pre-publication history for this paper can be accessed here:


